# Gender Difference in the Prognostic Role of Interleukin 6 in Oral Squamous Cell Carcinoma

**DOI:** 10.1371/journal.pone.0050104

**Published:** 2012-11-21

**Authors:** Chih-Jung Chen, Wen-Wei Sung, Yueh-Min Lin, Mu-Kuan Chen, Ching-Hsiao Lee, Huei Lee, Kun-Tu Yeh, Jiunn-Liang Ko

**Affiliations:** 1 Institute of Medicine, Chung Shan Medical University, Taichuang, Taiwan; 2 Institute of Medical and Molecular Toxicology, Chung Shan Medical University, Taichuang, Taiwan; 3 Department of Surgical Pathology, Changhua Christian Hospital, Changhua, Taiwan; 4 Department of Medical Technology, Jen-Teh Junior College of Medicine, Nursing and Management, Miaoli, Taiwan; 5 School of Medicine, Chung Shan Medical University, Taichuang, Taiwan; 6 Department of Otorhinolaryngology, Head and Neck Surgery, Changhua Christian Hospital, Changhua, Taiwan; 7 Department of Medical Oncology and Chest Medicine, Chung Shan Medical University Hospital, Taichuang, Taiwan; Innsbruck Medical University, Austria

## Abstract

**Background:**

Interleukin 6 (IL6) plays an important role in immunoregulation and tumorigenesis in human cancers. Oral squamous cell carcinoma (OSCC) is a malignant tumor of the oral cavity with a male predominant tendency and a poor clinical prognosis. Due to the relatively few cases in females, the gender difference of prognostic markers for OSCC is seldom discussed.

**Methods:**

In this study, we used immunohistochemical staining methods to investigate the associations between IL6 expression and the clinicopathological characteristics of OSCC. In addition, we collected 74 female and 263 male OSCC patients for evaluation.

**Results:**

High IL6 expression in tumor cells was significantly associated OSCC patient characteristics including female gender (P<0.001), high lymph node metastatic rate (P = 0.007), and poor tumor differentiation (P = 0.008). Tumor-expressed IL6 had prognostic role in male OSCC patients as defined by the log-rank test (P = 0.014), but not in female patients (P = 0.959). In male OSCC patients, high IL6 expression in tumor cells was associated with poor prognosis (P = 0.025) and a 1.454-fold higher death risk, as determined by Cox regression.

**Conclusions:**

High IL6 expression in tumor cells was therefore significantly associated with aggressive clinical manifestations and might be an independent survival predictor, particularly in male OSCC patients.

## Introduction

Oral squamous cell carcinoma (OSCC) is a common malignant tumor of the oral cavity and has a poor clinical prognosis [1]. In Taiwan, OSCC is the fourth cause of cancer-related death [2], [3] and its main etiologies include smoking, alcohol consumption, and betel quid chewing [4]. It is predominant in Taiwanese males, whereas the OSCC incidence in females is relatively low [4], [5].

Interleukin 6 (IL6), also known as B cell differentiation factor, is an immunoregulatory cytokine with biological functions of pro-inflammation, anti-inflammation, and angiogenesis. These responses are triggered by activation of the JAK tyrosine kinase family and then further stimulated by the MAPK, PI3K, or STAT signaling pathways [6]–[8]. IL-6 is released in response to infection, burns, trauma, and neoplasia [6]–[8] and can be produced by T lymphocytes, B lymphocytes, macrophages, endothelial cells, keratinocytes, and mesangial cells in normal tissues, as well as by hematologic neoplasms and human cancers [7]. It regulates the final differentiation of B cells into plasma cells and the proliferation or differentiation of cytotoxic T cells [6].

Increased IL6 expression in either serum, saliva, or tumor tissues has been noted in patients with colorectal cancer, breast cancer, lymphoma, hepatocellular carcinoma, pancreatic carcinoma, renal cell carcinoma, bladder cancer, and multiple myeloma and it has been implicated in tumor development [8]–[18]. Increased IL6 expression has therefore been proposed as a clinically relevant factor for poor prognosis in patients with a number of cancers, including colorectal cancer, non-small cell lung cancer, Hodgkin’s lymphoma, and renal cell carcinoma [9]–[11], [17]. In addition, IL6 gene polymorphisms are associated with increased cancer risk for colorectal carcinoma [19]. For these reasons, IL6 has become a therapeutic target and treatment with anti-IL6 antibody has resulted in complete inhibition of bone invasion of oral cancer cells *in vitro* and in an *in vivo* animal models [20]. Increased IL6 expression has also been reported in the serum and saliva of oral and oropharyngeal cancer patients [21]–[24].

Increased IL6 expression is thought to specifically promote tumor progression, angiogenesis, or bone invasion in head and neck cancer patients [20], [22], [23], [25]. However, the association between increased IL6 expression and clinical prognosis for head and neck cancer patients remains controversial [21], [26]–[28]. Some of the study populations are relatively small and rely on evaluation of IL6 levels in the serum or saliva rather than in tumor tissues. In addition, few female patients have been included in previous studies. The prognostic value of IL6 expression and its potential for clinical application between genders therefore still require further verification and validation.

In the present study, we investigated the prognostic value of IL6 in OSCC patients from a Taiwanese population, and purposely included female OSCC patients in order to evaluate their clinicopathological features.

## Materials and Methods

### Patients and Tissue Microarrays

A total of 337 patients were enrolled in this study. All had been diagnosed with OSCC by histopathological exam and underwent surgical resection at Changhua Christian Hospital between 2000 and 2006. No patient underwent preoperative radiotherapy, chemotherapy, or any other treatment. Formalin-fixed, paraffin-embedded tissue microarrays composed of 337 OSCC tissue cores were constructed as previously described [29]. The clinical data including sex, age, grade, smoking, alcohol consumption, betel quid chewing, T, N, and M stages, post-operative adjuvant therapy, and follow-up information including recurrence, living, or death were obtained from medical records and the cancer registry. This study was approved by the Institutional Review Board and the Ethics Committee of the Changhua Christian Hospital (IRB serial number: 111014) and Chung Shan Medical University Hospital (IRB serial number: CS11178).

**Table 1:** The clinicopathological characteristics by gender in 337 oral squamous cell cancer (OSCC) patients.

	Gender		
Parameters	Female (%)	Male (%)	P value
Smoking			
No	60 (85.7)	86 (33.9)	<0.001*
Yes	10 (14.3)	168 (66.1)	
Alcohol consumption			
No	64 (88.9)	120 (46.9)	<0.001*
Yes	8 (11.1)	136 (53.1)	
Betel quid chewing			
No	61 (88.4)	114 (49.6)	<0.001*
Yes	8 (11.6)	116 (50.4)	
Stage			
I	31 (41.9)	41 (15.6)	<0.001*
II	10 (13.5)	44 (16.7)	
III	8 (10.8)	20 (7.6)	
IV	25 (33.8)	158 (60.1)	
Stage			
I+II	41 (55.4)	85 (32.3)	<0.001*
III+IV	33 (44.6)	178 (67.7)	
Grade			
Well	7 (9.5)	43 (16.3)	0.312
Moderate	66 (89.2)	215 (81.7)	
Poor	1 (1.3)	5 (2.0)	
Death			
No	54(73.0)	106 (40.3)	<0.001*
Yes	20 (27.0)	157(59.7)	

Note: P-value determined by Pearson chi-square. *P<0.05.

### Analyses of IL6 Expression by Immunohistochemistry Staining (IHC Staining)

The OSCC tissues were embedded in paraffin and sectioned (4 µm). The sections were placed on coated slides, washed with xylene to remove the paraffin, and rehydrated through serial dilutions of alcohol, followed by washings with a solution of phosphate buffered saline, PBS (pH 7.2). Endogenous peroxidase activity was blocked with 3% H_2_O_2_. Antigen retrieval was performed by boiling in citrate buffer (10 mM) for 20 min. After incubation with the anti-human IL6 antibody (1∶100 dilution; sc-7920, Santa Cruz Biotechnology) for 20 min at room temperature and thorough washing (three times with PBS), the slides were incubated with a horseradish peroxidase (HRP)/Fab polymer conjugate for another 30 min. The sites of peroxidase activity were visualized using 3,3′-diamino-benzidine tetrahydrochloride as the substrate and hematoxylin as the counterstain. PBS was used instead of primary antibodies as a negative control. The stromal lymphocytes were used as the positive control. Cytoplasmic IL6 staining of cancer cells was regard as positive staining. For the purpose of eligibility, positive IL6 IHC was defined by greater than 10% positive cells [30]. The intensity of staining was scored as −, 1+, 2+, and 3+ for negative, weak, moderate, and strong staining, respectively. Expression of IL6 was assessed semiquantitatively by 2 pathologists (Dr. CJ Chen and Dr. KT Yeh), who scored coded sections independently based on the staining score. A final agreement was obtained for each score, even for discrepant immunostaining results.

**Figure 1:**
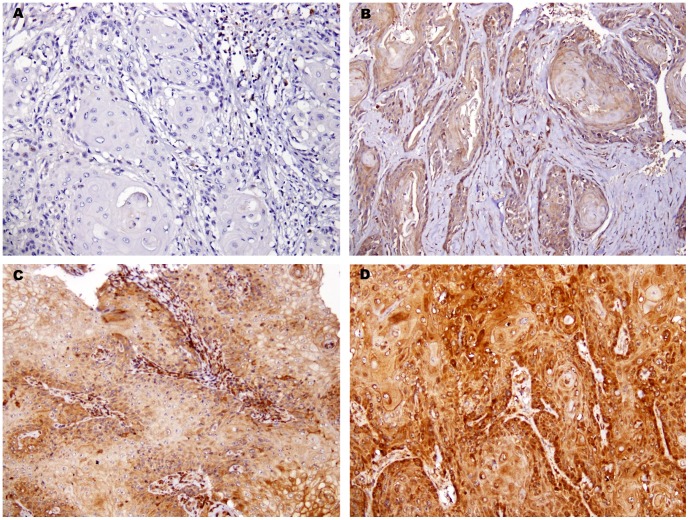
Representative immunohistochemical (IHC) staining patterns of tumor tissues for IL6 immunoreactivity in oral squamous cell carcinoma (OSCC). (A) Negative or barely conspicuous cytoplasmic staining (intensity = 0). (B) Weak cytoplasmic staining in OSCC (intensity = 1+). (C) Moderate cytoplasmic staining in OSCC (intensity = 2+). (D) Strong cytoplasmic staining in OSCC (intensity = 3+). (Immunohistochemistry: original magnification×200).

**Figure 2:**
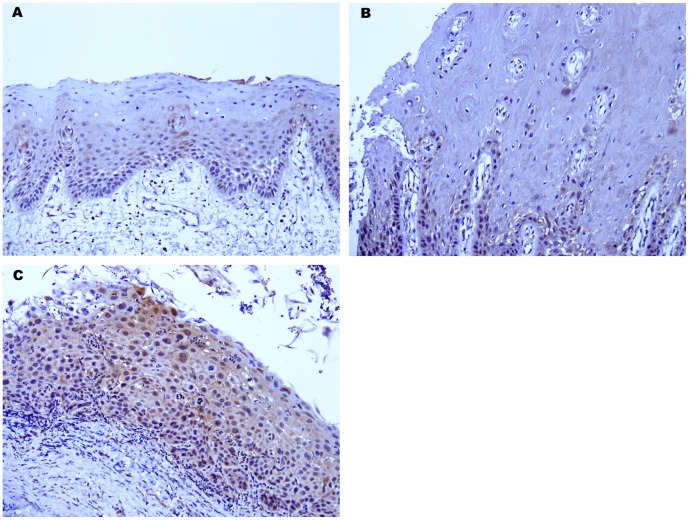
Representative IHC staining patterns for IL6 in normal oral epithelium (A) showing negative or barely conspicuous cytoplasmic staining (intensity = 0), hyperplastic oral epithelium (B) showing negative or barely conspicuous cytoplasmic staining (intensity = 0) and dysplastic oral epithelium (C) showing weak cytoplasmic staining (intensity = 1+). (Immunohistochemistry: original magnification×200).

### Statistical Analysis

All 337 cases were analyzed. The correlation of IL6 expression and clinicopathological parameters of OSCC were examined with Pearson chi-square test. The overall survival (OS) was defined as the time from the initiation of surgery to death. Relapse free survival (RFS) was defined as the time between date of diagnosis and date of local recurrence/distant metastasis. The distribution of OS was estimated using a Kaplan-Meier plot and the log-rank test. The prognostic significance of the variables was evaluated using the Cox regression model and hazard ratios. The variables in the model included gender, stage, T value, N value, M value, grade, and IL6 expression level. The analyses were performed using the Statistical Package for Social Sciences, Version 15.0 (SPSS, Version 15.0, Chicago, IL, USA), and *P*<0.05 (two-tailed test) was considered statistically significant.

**Table 2:** The association between tumor IL6 expression and clinical parameters in 337 oral squamous cell cancer (OSCC) patients.

		IL6^1^		
Parameters	Case No.	Low/Negative (%)	High (%)	P value
Age				
<56	194	129 (66.5)	65 (33.5)	0.680
≥56	143	92 (64.3)	51 (35.7)	
Gender				
Female	74	39 (52.7)	35 (47.3)	0.008*
Male	263	182 (69.2)	81 (30.8)	
Smoking				
No	146	89 (61.0)	57 (39.0)	0.080
Yes	178	125 (70.2)	53 (29.8)	
Alcohol consumption				
No	184	117 (63.6)	67 (36.4)	0.212
Yes	144	101 (70.1)	43 (29.9)	
Betel quid chewing				
No	175	104 (59.4)	71 (40.6)	0.028*
Yes	124	89 (71.8)	35 (28.2)	
Stage				
I+II	126	85 (67.5)	41 (32.5)	0.574
III+IV	211	136 (64.5)	75 (35.5)	
T value				
T1+T2	174	114 (65.5)	60 (34.5)	0.980
T3+T4	163	107 (65.6)	56 (34.4)	
N value				
N0	213	151 (70.9)	62 (29.1)	0.007*
N1+N2	124	70 (56.5)	54 (43.5)	
Distant metastasis				
M0	331	219 (66.2)	112 (33.8)	0.093
M1	6	2 (33.3)	4 (66.7)	
Grade				
Well	50	41 (82.0)	9 (18.0)	0.008*
Moderate+poor	287	180 (62.7)	107 (37.3)	
RFS^2^				
Dead/Relapse	201	129 (64.2)	72 (35.8)	0.420
Alive	124	85 (68.5)	39 (31.5)	
OS^3^				
Dead	177	110 (62.1)	67 (37.9)	0.163
Alive	160	111 (69.4)	49 (30.6)	

Note: ^1^Low/negative IL6: weak (1+) or negative (−) IL6 staining. High IL6: strong (3+) or moderate (2+) IL6 staining; ^2^RFS: relapse free survival; ^3^OS: overall survival.

P-value determined by Pearson chi-square. *P<0.05.

## Results

### Patient Characteristics

In total, 337 patients, including 263 males and 74 females, were analyzed in this retrospective study. The mean age of the patients was 56.0±11.6 years. The histological tumor type of all 337 patients was squamous cell carcinoma. There were 72 patients with stage I tumors, 54 patients with stage II tumors, 28 patients with stage III tumors, and 183 patients with stage IV tumors. Of these tumors, 50 were well differentiated, 281 were moderately differentiated, and 6 were poorly differentiated. The OS time ranged from 0.1 to 9.0 years, with a mean survival time of 4.0±2.8 years and a median survival time of 3.9 years. Adjuvant therapy was administered according to individual considerations. There were 324 patients with smoking history, 328 patients with alcohol drinking history, and 299 patients with betel quid chewing history according to the chart records. The rest of the patients had missing or unrecorded data in their charts.

**Table 3:** Univariate and multivariate analysis of the influence of various parameters on overall survival in oral squamous cell cancer (OSCC) patients (337 cases).

	Univariate analysis			Multivariate analysis^#^		
Parameter	HR^1^	95% CI^2^	P	HR^1^	95% CI^2^	P
Gender						
Female	1.000	Referent	<0.001*	1.000	Referent	0.002*
Male	2.402	1.508–3.828		2.086	1.300–3.346	
Stage						
I+II	1.000	Referent	<0.001*	1.000	Referent	0.001*
III+IV	1.949	1.407–2.700		1.741	1.251–2.423	
T value						
T1+T2	1.000	Referent	<0.001*	1.000	Referent	0.205
T3+T4	1.862	1.383–2.508		1.314	0.862–2.004	
N value						
N0	1.000	Referent	<0.001*	1.000	Referent	0.007*
N1+N2	1.887	1.387–2.549		1.645	1.148–2.358	
M value						
M0	1.000	Referent	0.025*	1.000	Referent	0.053
M1	2.777	1.138–6.777		2.432	0.990–5.971	
Grade						
Well	1.000	Referent	0.030*	1.000	Referent	0.010*
Moderate+poor	1.696	1.054–2.730		1.876	1.163–3.025	
IL6						
Low/Negative	1.000	Referent	0.138	1.000	Referent	0.036*
High	1.259	0.929–1.706		1.389	1.021–1.890	

Note:^1^HR: hazard ratio; ^2^CI: confidence interval. Statistical significance was defined as * P<0.05 ^#^Adjusted for gender and stage.

**Table 4:** Multivariate analysis of the influence of various parameters on overall survival (OS) in oral squamous cell cancer (OSCC) patients according to IL6 expression (337 cases).

Group	Case No.^1^	OS			
		Median surviral(year)^1^	HR^2^	95% CI^3^	P
Gender					
Female	35/39	8.8/>9.0	1.023	0.424–2.469	0.959
Male	81/182	2.6/5.3	1.493	1.078–2.068	0.016*
Stage					
I+II	41/85	7.5/8.0	1.243	0.704–2.195	0.453
III+IV	75/136	2.7/4.5	1.253	0.874–1.796	0.220
Differentiation					
Well	9/41	6.4/>9.0	1.577	0.517–4.809	0.423
Moderate+poor	107/180	5.2/5.3	1.163	0.846–1.599	0.352

Note:^ 1^IL6 high/IL6 (low/negative);^ 2^HR: hazard ratio; ^3^CI: confidence interval. Statistical significance was defined as *P<0.05.

The clinicopathological characteristics of female and male OSCC patients are shown in Table 1. Female patients tended to have habits of no smoking, no betel quid chewing and no alcohol consumption. In addition, female patients showed a tendency to have with less advanced cancer stages and better clinical prognosis.

**Figure 3:**
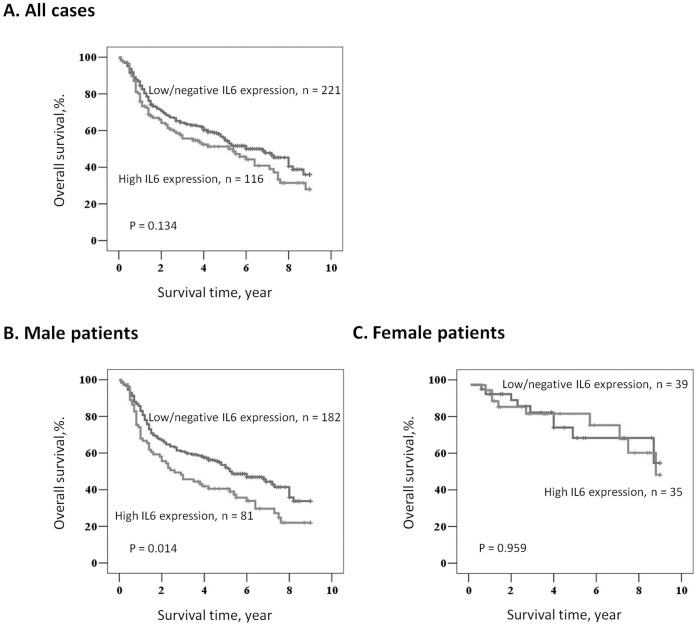
Analysis of the overall survival of patients with varying immunoreactivities of tumor tissues for IL6 expression. (A) Kaplan-Meier survival curves for OSCC patients who were classified with either low/negative (weak (1+) or negative (−) IHC positivity) or high IL6 (strong (3+) or moderate (2+) IHC positivity) expression. The IL6 status was not significantly associated (log-rank, P = 0.134) with patient survival. (B) In male OSCC patients, the IL6 status was significantly associated (log-rank, P = 0.014) with patient survival. (C) In female OSCC patients, no significant association was found for IL6 expression and patient survival (log-rank, P = 0.959).

### IL6 Expression in OSCC Tumors and Adjacent Tissues

IL6 was expressed in the cytoplasm of cancer cells (Fig. 1). The expressions were diffused or patchy within the tumor tissues and were also evident in the background lymphocytes. Of the 337 tumors, 226 (67.1%) displayed IL6 cytoplasmic staining. The IL6 expression was classified by staining density as high IL6 expression with strong (3+) or moderate (2+) IHC positivity and low/negative IL6 expression with weak (1+) or negative (−) staining. This revealed 116 tumors (34.4%) belonging to a high IL6 expression group and 221 tumors (65.6%) belonging to a low/negative IL6 expression group (Fig. 1).

We also analyzed the IL6 expression of the tissues adjacent to the invasive OSCC in whole-mount sections. We observed that normal oral epithelium or hyperplastic oral epithelium displayed negative or faint/barely perceptible cytoplasmic staining of IL6 (Fig. 2). However, dysplastic oral epithelium showed weak cytoplasmic staining of IL6 (Fig. 2).

Table 2 shows the relationship between clinicopathological factors and IL6 expression of tumor cells. When IL6 expression was classified using a 2-tier grading system, high IL6 expression in tumor cells was significantly associated with a high rate of lymph node metastasis (P = 0.007) and poor tumor differentiation (P = 0.008). High IL6 expression was associated with female gender (P = 0.008) and inversely associated with betel quid chewing (P = 0.028). However, no significant association was found between IL6 expression and patient age, smoking, alcohol consumption, tumor size, distant metastasis, tumor stage, RFS, or OS.

### Prognostic Value of IL6 Expression of Tumor Cells in OSCC

The univariate analyses performed using the Cox proportional hazard regression model identified that male gender (P<0.001), advanced clinical stages (P<0.001), larger tumor size (P<0.001), positive lymph node metastasis (P<0.001), distal metastasis (P = 0.025), and poorer tumor differentiation (P = 0.030) were correlated with poor OS (Table 3). High IL6 expression was not significantly correlated with poor OS (P = 0.138). However, in the multivariate analysis model, a significantly poorer OS rate was seen for OSCC patients with high IL6 expression than with low/negative IL6 expression (P = 0.036; Table 3). The multivariate analysis revealed that only male gender (P = 0.002), advanced clinical stage (P = 0.001), positive lymph node metastasis (P = 0.007), and poor tumor differentiation (P = 0.010) were correlated with poor survival. When we further grouped the patients according to gender, we found that the male patients with high IL6 expression had shorter median survival years than those with low/negative IL6 expression (2.6 years vs. 5.3 years) (P = 0.025; Table 4). The hazard ratio for OS in males was 1.454 (95% confidence interval = 1.049–2.015) for high IL6 expression compared to low/negative IL6 expression as determined by multivariate analysis.

The Kaplan-Meier survival curves demonstrated no significant difference in OS between patients with high IL6 expression [strong (3+) or moderate (2+) IHC positivity] and patients with low/negative IL6 expression [weak (1+) or negative (−) IHC positivity], as defined by the log-rank test (P = 0.134; Fig. 3A). When we subdivided patients by gender, we also found that high IL6 expression was significantly associated with poor OS in male patients (P = 0.014; Fig. 3B). However, no significant association was found between IL6 expression and OS in female patients (P = 0.959; Fig. 3C).

## Discussion

In this study, we found that high expression of cytosolic IL6 in OSCC tumors is correlated with female gender, a high rate of lymph node metastasis, and poor tumor differentiation. Therefore, high IL6 expression could be an independent prognostic factor in OSCC patients in Taiwan, especially in male patients. The IL6 expression was higher in OSCC cancer cells than in normal tissues and IL6 appeared to be involved in the tumorigenesis of OSCC.

Other studies have been initiated to investigate the clinical significance of IL6 expression in tumor cells, rather than measuring serum or saliva IL6 levels, but the results are still controversial [21], [26]–[28]. For example, Duffy SA et al. demonstrated that high serum IL6 level in 444 OSCC patients, including 350 male and 94 female patients, was associated with a significantly lower OS [26]. High serum IL6 expression was also associated with age, smoking status, and tumor stage. No gender difference was found for IL6 expression. Wang YF et al. evaluated tissues from 86 OSCC patients using an in situ hybridization method to detect IL6 mRNA expression levels and demonstrated that the presence of IL6 mRNA transcripts in the tumor cells was inversely associated with distant metastasis and lymph node involvement [27]. In contrast, elevated levels of IL6 mRNA transcripts were also shown to predict better survival in OSCC patients [27]. We have long suspected that high IL6 expression might be associated with a worse prognosis (P = 0.036, by multivariate analysis, Table 3) However, this association was not found when we used univariate analysis (P = 0.138) or when we examined Kaplan-Meier survival plots (P = 0.134). We ultimately found that high IL6 expression was associated with poor prognosis in male OSCC patients. This discrepancy might have resulted from the use of different study populations or methodologies, or from different OSCC patient etiologies including smoking, alcohol consumption, and betel nut chewing.

In the present study, we found an association between IL6 expression and lymph node metastatic rate in OSCC patients, which is consistent with the previous findings of Shinriki S et al. and Nagata M et al. [31], [32]. In colorectal and breast cancers, high IL6 expression was also associated with a high rate of lymph node metastasis [9], [11]. This may result from promotion of angiogenesis mediated by IL6 expressed in tumors [31], [32]. In addition, high IL6 expression was inversely associated with betel nut chewing, but was not associated with smoking or alcohol consumption. We suspected that the high IL6 expression level in tumors from female patients, who did not chew betel quids, indicated a differential impact of IL6 on OSCC in different study populations.

Previous reports demonstrated a high expression of IL6 in OSCC tumor cells or premalignant lesions, including dysplasias, when compared with non-tumor oral epithelium [21], [22], [27]. In addition, IL6 could induce the *in vitro* growth of oral squamous cells. These findings suggested that IL6 plays an important role in the development of oral cancer [21], [27]. The IHC methods used in the present study frequently revealed a weak and diffuse expression of IL6 in dysplastic oral epithelium. Normal mucosal epithelium or hyperplastic epithelium showed negative or barely conspicuous IL6 expression. Our findings were consistent with those of previous studies [21] and we found similar IL6 expression patterns during progressive cell transformation in OSCC based on histological observations.

OSCC is mainly found in males in Taiwan and the high male/female ratio has been noted in previous epidemiology reports [4], [5]. The oral cancer incidence rates in female patients are very low in Taiwan, which means that we have limited knowledge about female OSCC. Our study is the first to report that a high IL6 expression in OSCC seems to have an important bearing in a particular sup-group of OSCC patients; namely, male OSCC patients. In this group, high IL6 expression seemed to be associated with poor prognosis when compared with female OSCC patients. We have previously speculated on possible causes that lead to the different impact of IL6 observed between male and female patients and an impact of human papilloma virus (HPV) infection has been suspected [33]–[35]. We therefore analyzed the HPV16 E6 in our cases and found: (1) No association of HPV16 E6 expression with male and female genders (P = 0.258, data not shown); (2) No association of HPV16 E6 expression with IL6 expression (P = 0.491, data not shown); (3) No association of HPV16 E6 with OS in univariate and multivariate Cox regression model analysis (P = 0.818 and P = 0.608, respectively, data not shown); and (4) No association, by multivariate analysis, of HPV16 E6 status with OS in OSCC patients according to IL6 expression. For HPV negative patients, no difference is seen in survival between high and low IL6-expressed OSCC patients (P = 0.182, data not shown). For HPV positive patients, no difference is again seen in survival in high and low IL6-expressed OSCC patients (P = 0.445, data not shown). In addition, we also found a similar proportion of HPV infection in both male and female patients (male: 21.4% and female: 27.8%) and HPV infection status shows no statistical significance between genders. Therefore, we ultimately concluded that HPV might not influence IL6 expression. This result suggested the existence of key differences in IL6 effects and clinical etiologies in female and male OSCC patients. The different roles played by tumor-expressed IL6 in males and females need further investigation.

Our study had some limitations. IL6 is an immunoregulatory cytokine and has been reported to promote tumor progression in human cancers [6]–[8]. We proposed that activation of the STAT3 pathway, angiogenesis, or bone invasion induced by IL6 could contribute to the poor prognosis of patients expressing high levels of IL6 [7], [20], [21]. However, we were limited in our ability to demonstrate the mechanism underlying the IL6 effects observed in our OSCC specimens. We could not use the tissue arrays or IHC to evaluate whether differences in microvascular density were present in the groups expressing different levels of IL6. Both cytosolic and secretory forms of IL6 are found in cells and in tissue microenvironments, and IL6 can be produced by tumor cells as well as by some immune cells or normal tissues [6], [7]. In addition, the IHC staining method does not allow detection of serum or saliva IL6 levels, so IHC was used as a semi-quantitative method to detect the protein levels in the tumor cells. The biological significance of IL6 expression in the OSCC specimens may be different from IL6 levels determined in serum or tissue specimens or detected with different methodologies.

In conclusion, this is the first report to show a difference in IL6 expression in female and male OSCC patients and to show a different prognostic role of IL6 between genders. Further validation studies looking at IL6 expression in larger cohorts of female and male patients should be conducted to characterize the clinical relevance of IL6 expression. We also identified that a high IL6 expression in tumor cells was correlated with poor OS. This high IL6 expression could be an independent prognostic marker in OSCC patients, which suggests that IL6 might be important as a potential therapeutic target in OSCC treatment.
